# Influenza vaccination from the perspective of health care workers at university hospitals

**DOI:** 10.1371/journal.pone.0288470

**Published:** 2023-07-21

**Authors:** Dominika Rykowska, Tomasz Sobierajski, Magdalena Okarska-Napierała, Monika Wanke-Rytt, Ernest Kuchar

**Affiliations:** 1 Department of Pediatrics with Clinical Assessment Unit, Medical University of Warsaw, Warsaw, Poland; 2 Faculty of Applied Social Sciences and Resocialization, University of Warsaw, Warsaw, Poland; American University of Beirut Medical Center, LEBANON

## Abstract

**Background:**

The influenza vaccination rate of healthcare workers (HWs) in Poland is low. Before implementing methods for promoting influenza vaccination, it is crucial to identify attitudes towards vaccination. We aimed to examine the knowledge and attitudes towards influenza vaccination of HWs at university hospitals. Moreover, we evaluated the incentives for getting influenza vaccination among HWs.

**Methods:**

From September 2020 to October 2020, we surveyed HWs in one children’s hospital and two adults’ hospitals in Warsaw (Poland). We included only fully and correctly completed surveys into final analysis.

**Results:**

A total of 950 questionnaires (85% women, 45% <40 years old, 33% physicians and 48% nurses, 56% working in a children’s hospital) were evaluated. Of all HWs, 25% declared they were vaccinated and 54% planned to get vaccinated in the next season. We have analyzed attitudes towards influenza vaccination and motivations to get vaccinated.

**Conclusions:**

Among HWs in academic hospitals, males, people <40 years old, physicians and those working in children’s hospital are more likely to get vaccinated and their attitudes towards influenza vaccination are more positive. Of those less likely to get vaccinated, people >40 years old and nurses could be effectively persuaded by free and on-site influenza vaccination. Moreover, free access to vaccination is the strongest motivator for vaccination among all HWs. The attitudes towards mandatory influenza vaccination differ sharply among HWs–while physicians are ready to accept it, nurses are not.

**Trial registration:**

ClinicalTrials.gov Identifier: NCT04569019.

## Introduction

The influenza virus is among the most common causes of lower respiratory tract infections. Every year from 5% to 10% of the adult global population and from 20% to 30% of the pediatric global population have a flu infection [1]. It is a disease of sudden onset usually characterized by fever, cough, muscle and joint pain, malaise, and headache, but severe complications, including death, are not uncommon. As shown in the World Health Organization (WHO) reports and Global Burden of Diseases Study from 2017, approximately 8.2 million people suffers from severe influenza infection resulting in 145,000 to 650,000 deaths yearly [1, 2]. More than 50% of the fatal outcomes are seen in adults older than 70 years old [2]. In children less than 5 years old the epidemiological burden of influenza infection was estimated for 10.1 million with up to 34800–105000 deaths annually [3, 4]. Among healthcare workers (HWs), the risk of influenza is twice as high as in general population [5].

Seasonal vaccinations, hands washing and wearing face masks are the most effective, scientifically proven methods for preventing influenza infection and its complications [6]. WHO recommends seasonal influenza vaccinations for all the population, especially for high-risk individuals: pregnant women at any stage of pregnancy, children aged between 6 months and 5 years, elderly individuals (aged more than 65 years), individuals with chronic medical conditions, including immunocompromised [1]. Moreover, HWs taking care of those high-risk groups should also be vaccinated [7, 8]. The value of seasonal flu vaccination is broader than self-protection. Increasing the influenza vaccination rate is an evidence-based public health goal, as the vaccination has not only personal but also economical (economic models, cost effectiveness), allocative (accessibility and equity in vaccination access), and social (well-being of the population and indirect protection for the community and vulnerable groups) values [9]. Flu vaccination protects not only HWs but also improves patient protection [9–15].

Though the flu vaccination has a substantial value, the vaccination rates in HWs are low. In Europe, according to the European Centre for Disease Prevention and Control (ECDC) report, in the 2016–2017 season, the average vaccination uptake among HWs was 30%, with the highest rates in England– 63.2% and lowest in Italy– 15.6% [16]. An international literature review by Guillari et al. identified barriers to influenza vaccine uptake among HWs. Not considering to be at risk of influenza infection, fear of acquiring influenza from vaccination itself, declaring no time for getting the flu shot, fear of the vaccination side effects and disbelieve in vaccine effectiveness were common reasons in many countries, including Poland, for declining influenza vaccination by HWs [17].

As mentioned, WHO recommends seasonal influenza vaccinations for HWs, and there is an ongoing discussion in the literature about mandatory vaccinations among HWs [18–20].

In Poland, there has been a low acceptance of influenza vaccination for years, with the vaccination rate of 6% in the general population [21].

The main objective of our study was to examine the attitudes towards influenza vaccination of medical and non-medical staff at university hospitals. Moreover, we evaluated the incentives for getting influenza vaccination among HWs.

To our knowledge, it is the first study conducted on a large group of university hospital staff on influenza vaccination in Poland.

## Materials and methods

The Ethics Committee of Warsaw Medical University in Poland approved the study (No. AKBE/118/2020).

### Study design

The survey was conducted among employees of the University Clinical Center of the Warsaw Medical University (UCK WUM), a complex of three teaching hospitals. Two of them are hospitals for adult patients and one is a children’s hospital. In addition, UCK WUM is responsible for educating students at all university faculties, medical residences, and comprehensive health promotion.

### Organization of vaccination programs for HWs

In Poland, influenza vaccination for the general population is optional and not funded. However, vaccines can be free of charge for specific social groups or clinical situations.

The organization of influenza vaccination for HWs changes in each season.

In the 2019/2020 season, influenza vaccination for HWs was neither obligatory nor funded. There was no vaccination program in the UCK WUM hospitals.

In the 2020/2021 season, UCK WUM purchased a vaccination pool in October and offered it free of charge to all willing hospital employees. In addition, vaccination centers on UCK WUM grounds were organized, where employees could get vaccinated during working hours. The information about the vaccination possibility was distributed by internal e-mails, and secretaries collected lists of persons willing to get vaccinated and set a date of vaccination for each department in the vaccination center.

### Vaccination promotion campaign in the children’s hospital

In the 2020/2021 season two authors of this study undertook an intensive vaccination promotional campaign in the pediatric UCK WUM hospital. MWR, as a member of the Infection Control Team, came up with the idea of mobile vaccination clinic. DR implemented the mobile vaccination clinic and, in cooperation with head nurses, arranged a vaccination schedule in each department so that all employees knew when to come for vaccination. If someone was absent, they could get vaccinated anytime during working hours in the Department of Pediatrics, where MWR and DR worked. This way, the procedure of vaccination arrangements was more "by-walk-in" rather than "by-appointment" as in the two other hospitals. Furthermore, random face-to-face or telephone conversations promoting vaccination were conducted by MWR and DR. Information posters, including details of influenza vaccination availability and DR telephone number, were hanged in the most visible spots in the hospital.

### Sample size and characteristics

A total of 1233 hospital employees took part in the survey. Only wholly and correctly completed questionnaires, in the number of 950, were included in the final analysis. The respondents, whom we collectively defined as HWs, were divided into three occupational groups: physicians, nurses, and other hospital employees (paramedics, pharmacy staff, laboratory staff, radiology and electroencephalography technicians, psychologists, medical registrars, dieticians, physiotherapists, administrative staff).

### The questionnaire

The survey questionnaire consisted of three parts. The first was metric questions about sex, age, occupation and the type of hospital. The second covered questions about planned or completed influenza vaccinations and direct contact with patients, including immunocompromised ones. In the third part, we asked respondents to evaluate specific attitudes and issues related to influenza vaccination, such as whether vaccination is an ethical obligation for those who come into contact with patients, whether vaccination protects against post-influenza complications, whether influenza vaccination is essential in the situation of the prevailing COVID-19 pandemic, whether vaccination should be made mandatory among medical staff and hospital employees, or whether hospital employees need to expand their knowledge of influenza vaccination. The English translation of the whole survey is included in the S1 File. All questions in the questionnaire were single-choice questions. The questions in the parts one and two were either dichotomous (“yes” or “no” answers), or alternative questions (“yes” or “no” or “do not know”). In the part three, a seven-point numerical scale was prepared for each question, based on a Likert scale, where one end of the scale meant "strongly disagree" and the other meant "strongly agree" [22]. The questionnaire used in the study was explicitly prepared for this study. Before implementing the proper survey, the questionnaire was externalized to check its quality and usability. For this purpose, a pilot study was conducted on 23 randomly selected hospital employees. None of those participating in the pilot study raised any objections regarding the questionnaire.

### Data collection

The survey was conducted between September and October 2020. Participation in the survey was voluntary and the data collected were anonymized. The survey was implemented using the PAPI technique (*Pen and Paper Interview*). After informed consent, participants filled the survey by themselves.

### Statistical analysis

All statistical analyses were performed using IBM SPSS Statistics 28.0.1.1. We calculated the sample size assuming the margin of error to be no more than 5% at the 95% confidence level. The UCK WUM had 8421 employees at the time of the survey, so the minimum required sample was 367 respondents. A descriptive analysis was conducted to describe the sample, and the results are presented as frequencies and percentages. Quantitative and categorical variables were described with the methods of descriptive statistics. The scales in the questionnaire were validated using Cronbach’s alpha test. Normality was calculated using Shapiro–Wilk tests. Finally, the chi-square or Kruskall-Wallis tests were used to compare the study groups. Figures were created with the use of R Core Team (2021). R: A language and environment for statistical computing. R Foundation for Statystical Computing, Vienna, Austria.

## Results

### Sociodemographic characteristics

At the time of the study 8421 people were employed in the three teaching hospitals: 7086 physicians and nurses and 1335 other hospital employees. Of those 762 (11%) physicians and nurses, and 188 (14%) other employees participated in the survey. Sociodemographic characteristics of the study group are presented in Table 1.

**Table 1:** Sociodemographic characteristics and declarations concerning previous and future influenza vaccination of the study group.

Sociodemographic characteristics	N = 950 n (%)
**Sex**	
**Female**	807 (84.9%)
**Male**	143 (15.1%)
**Age [years]**	
**18–40**	427 (44,95%)
**>40**	523 (55,05%)
**Occupation**	
**Physician**	306 (32.2%)
**Nurse**	456 (48.0%)
**Other**	188 (19.8%)
**Hospital type**	
**Adults’ hospital**	422 (44.4%)
**Children’s hospital**	528 (55.6%)
**Direct contact with patients**	
**Contact with patients**	866 (91.2%)
**Contact with immunocompromised**	612 (64.4%)
**No contact with patients**	84 (8.8%)
**Declared vaccination status in 2019/2020 season**	
**Vaccinated**	240 (25.3%)
**Not vaccinated**	700 (73.7%)
**Does not remember**	10 (1%)
**Declared will for vaccination in 2020/2021 season**	
**Yes**	512 (53.9%)
**No**	337 (35.5%)
**Does not know**	101 (10.6%)

### Getting vaccinated against influenza

#### Declared previous vaccination during 2019/2020 season

Two hundred forty (25.3%) of HWs declared that they had been vaccinated against influenza during 2019/2020 season, whereas 700 (73.7%) had not been vaccinated (Table 1).

Physicians got vaccinated more often than nurses and non-medical employees (respectively: 49.7%, n = 152 v. 13.4%, n = 61 v. 14.4%, n = 27, p <0.001). Moreover, males, people <40 years of age and those working in children’s hospital were more likely to declare they got vaccinated.

Among those who had contact with immunocompromised patients 118 (53.4%) of the physicians, 46 (14.2%) of the nurses and 10 (15.2%) of the others declared they were vaccinated. Working with immunosuppressed was associated with higher vaccination coverage only in the case of physicians (53.4% v. 40%, p = 0.007). Moreover, HWs working in the children’s hospital were more commonly vaccinated if they cared for immunosuppressed (40.1%, n = 80 v. 22.6% n = 74, p<0,001) as opposed to those working in adults’ hospitals (14,8%, n = 42 v. 15.1%, n = 21, p = 0.002). The detailed results concerning declared previous vaccination with respect to working with immunosuppressed patients are presented in S1 File.

#### Declared will to get vaccinated in 2020/2021 season

In the 2020/2021 season, at the peak of the COVID-19 pandemic, when influenza vaccination was explicitly recommended, 512 (53.9%) of the respondents intended to get vaccinated and 337 (35.5%) did not intend to get vaccinated (Table 1). Regarding declaration of vaccination, males, people <40 years old, physicians, and people working in the children’s hospital were significantly more likely to declare the will to get vaccinated (p < 0.001).

**Fig 1:**
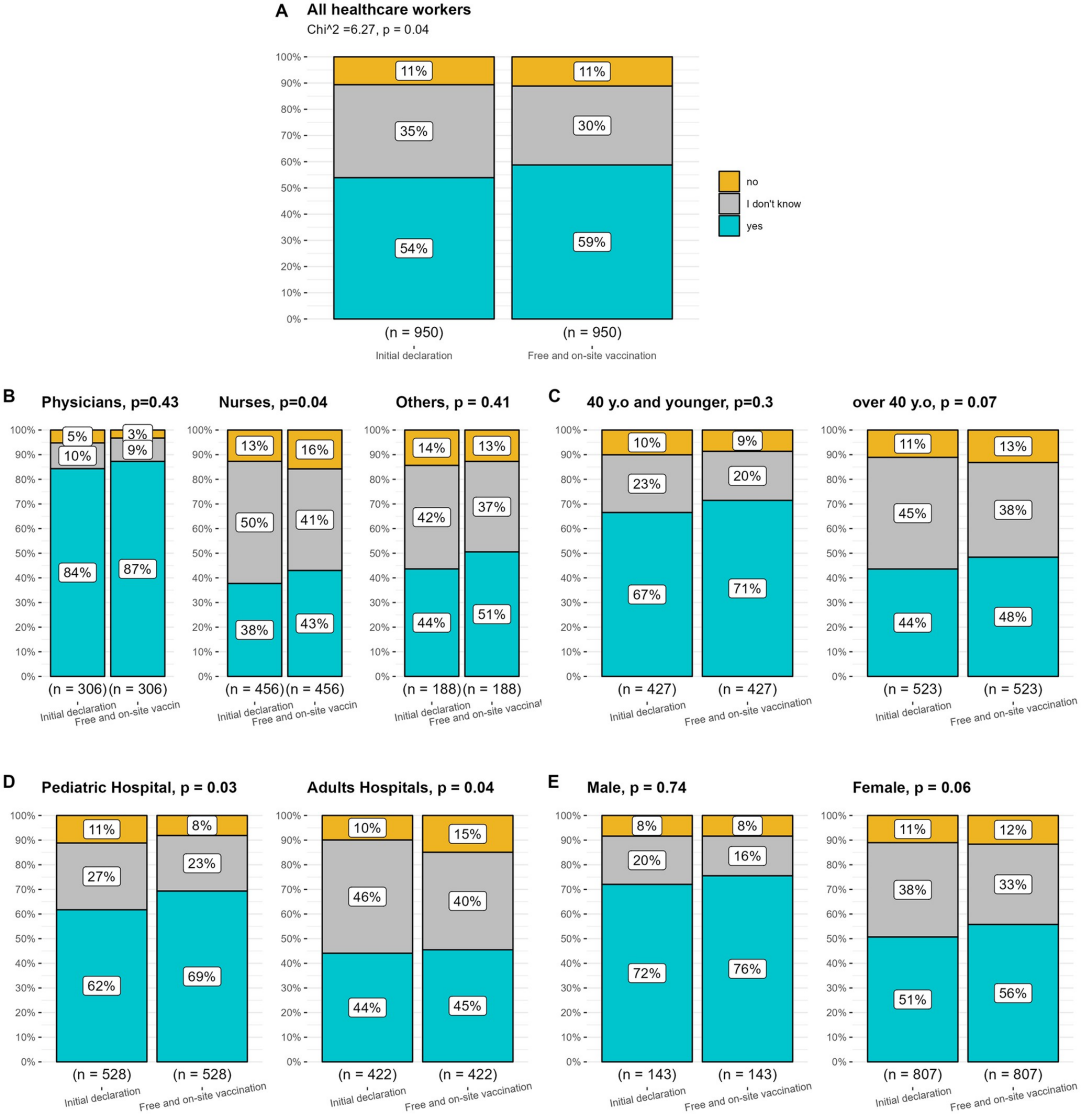
Change of declared will to get vaccinated if the influenza vaccines were free and on-site. (N = 950).

*How would free and on-site vaccination change declared will to get vaccinated*?. When we asked whether our respondents would get vaccinated if the influenza vaccine was free and on-site, we noted a significant change of mind among all HWs (Fig 1, p = 0.04). Overall, the percentage of HWs willing to get vaccinated was higher if the vaccine would be free and on-site. Considering specific groups, this change of mind was statistically significant for nurses, persons over 40 years old and both–HWs from children’s and adults’ hospitals. This is illustrated in Fig 1.

### Motivation for vaccination

**Fig 2:**
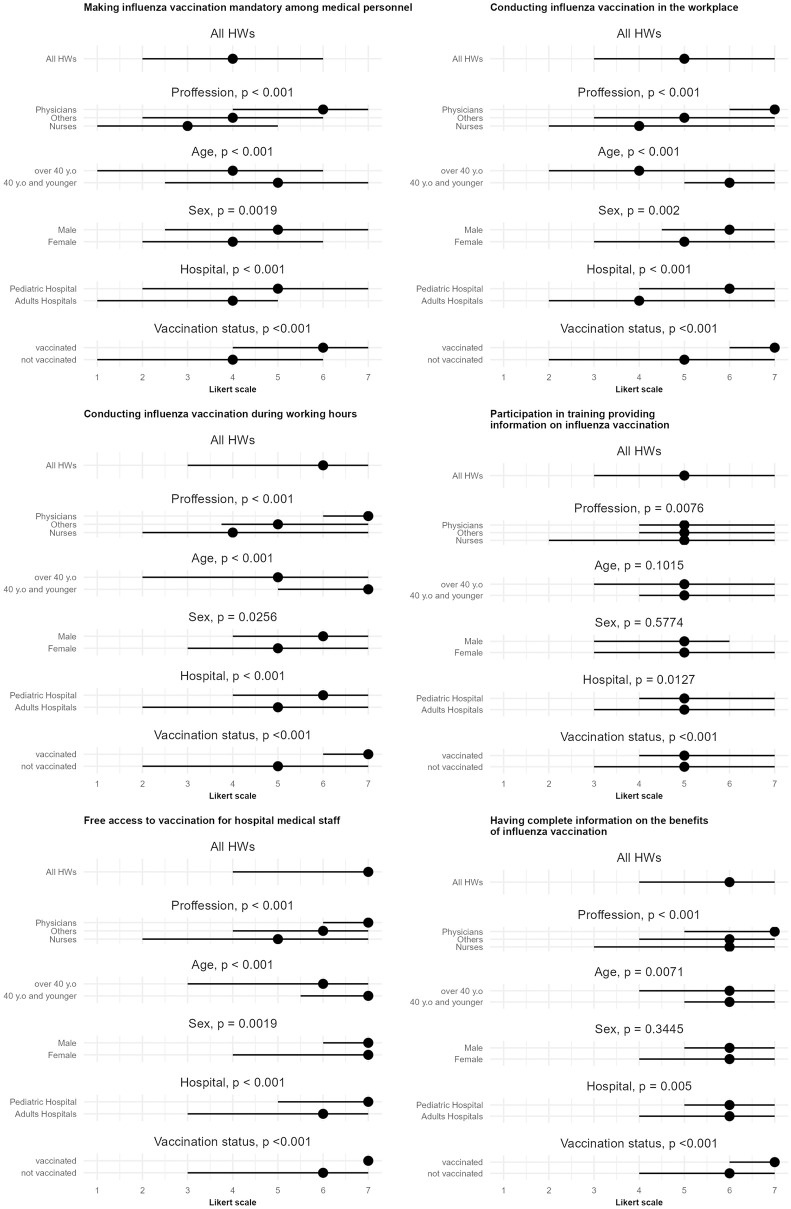
Motivation to get vaccinated against influenza (N = 950). Lines indicate 1st and 3rd quantile. Dots show median value. Note. Data based on a Likert scale, where 1 meant "totally disagree" and 7 meant "totally agree".

For all the statements concerning motivation to get vaccinated against influenza, male respondents, those <40 years old, physicians and employees of children’s hospital were more likely to agree than disagree (Fig 2).

In a situation where influenza vaccination among medical personnel would be made mandatory, 236 (24,9%) of respondents would be motivated to get vaccinated, but almost the same proportion of people 217 (22.8%) would definitely be discouraged. In a situation where the flu vaccination would be conducted at the workplace, it would definitely convince 369 (38.8%) to get vaccinated, while it would definitely not convince 125 (13.2%) of respondents. If the vaccination was conducted during working hours, it would definitely convince 408 (42.9%) respondents while it would definitely not convince 135 (14.2%) of respondents. Participation in a training course providing information on influenza vaccination would definitely encourage 270 (28.4%) to get vaccinated, while it would definitely not encourage 106 (11.2%) of respondents. Free access to immunization for hospital staff would definitely convince 500 (52.6%) of respondents, while definitely not– 100 (10.5%) of respondents. Complete information about the benefits of influenza vaccination would definitely encourage 427 (44.9%) of respondents to get vaccinated, while it would definitely not encourage 82 (8.6%) of respondents.

Making influenza vaccination mandatory, conducting vaccination at the workplace, conducting vaccination during working hours, free access to immunization for hospital staff, and having complete information about the benefits of influenza vaccination would be the most persuasive to get vaccinated for physicians and the least persuasive for nurses (*p* <0.001). The detailed results concerning incentives for getting influenza vaccination among HWs are presented in S1 File.

In juxtaposing the responses of vaccinated persons with those of unvaccinated persons against influenza in the 2019/2020 season from the perspective of individual motivators that could influence vaccination, the strength of each motivator to get vaccinated was more significant for vaccinated persons (Fig 2).

### Staff knowledge and attitudes toward influenza vaccination

#### Staff knowledge and attitudes toward influenza vaccination

**Fig 3:**
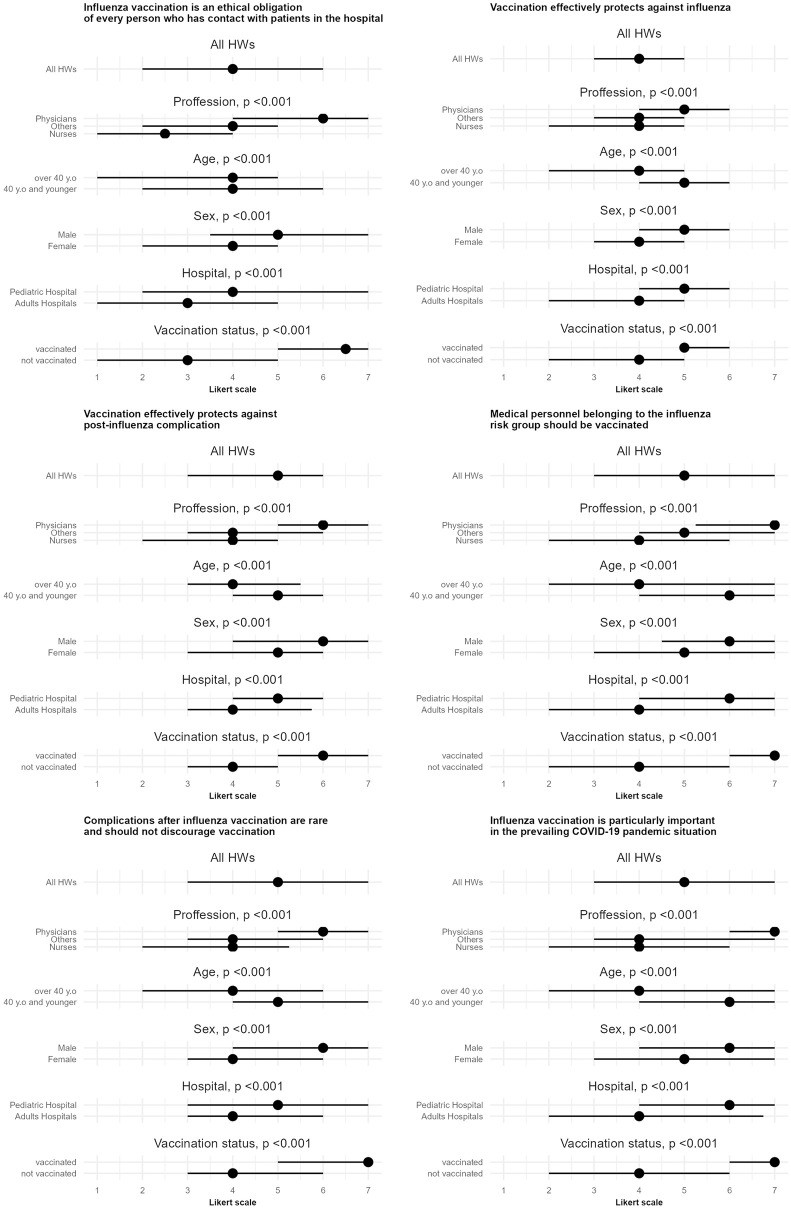
Knowledge and attitudes of hospital staff to individual claims about influenza vaccination (N = 950) Lines indicate 1st and 3rd quantile. Dots show median value. Note. Data based on a Likert scale, where 1 meant "totally disagree" and 7 meant "totally agree".

For all the statements concerning knowledge and attitudes towards influenza vaccination male respondents, those younger than 40 years old, physicians, and employees of children’s hospital were more likely to agree than disagree (Fig 3).

## Discussion

In our study, we examined influenza vaccination coverage, the attitudes and motivation toward vaccination among medical and non-medical staff at university hospitals in Poland.

Of all HWs, 25.3% were vaccinated against influenza. It is an average value when compared to influenza vaccination rate among HWs in other European countries [16, 23]. However, according to ECDC Technical Report, vaccination coverage of Polish HWs in 2014–2015 was only 5% [24]. In a Polish nation-wide study conducted at the same time as ours (2020/2021 season), 5.5% respondents declared they were vaccinated against influenza and 13.4% planned to get vaccinated, but finally 6% got vaccinated [21]. Thus, our results may reflect increased vaccination coverage of HWs in Poland when compared to both: general population and HWs from the past. However, our study group was not representative of all Polish HWs–we recruited only people from university hospitals, and over half of them worked in a pediatric hospital. Similarly, in another Polish study including a group of pediatricians and general practitioners administering vaccines to children, influenza vaccination acceptance was high [25]. In Poland, vaccine education and administration are the domain of pediatricians and family doctors. This is a likely explanation of that in our study working in a pediatric hospital was a factor significantly associated with being vaccinated and motivated to vaccinate in the future. Moreover, the promotional measures introduced in our pediatric hospital in the 2020/21 season could have influenced our results as well. Schumacher et al. proved that combining an educational program with on-site-vaccination is more effective than implementing on-site-vaccination only [26]. The "walk-in" vaccination procedure could have also raised the number of HWs vaccinated in our children’s hospital, as it was found to be effective intervention in other studies [27]. Unvaccinated HWs put patients at risk of hospital-acquired infection, and a high vaccination rate among HWs helps protect the patient from influenza, which represents a component of patient-centered health system, being a part of a value-based healthcare approach [28].

Surprisingly, males were more likely to get vaccinated than females, which is in contrast to other studies in both general populations and HWs [29–31]. Relatively small subgroup of males in our study could have influenced this result.

In our study, we found a significant correlation between vaccination attitudes and occupation, with physicians being more likely to get vaccinated than nurses and other HWs. To our knowledge, it was the first study to assess these differences in Poland, but our results are consistent with the findings from other countries. In Germany, significant disparities in vaccination coverage among physicians and nurses were found [32]. A comprehensive literature review of influenza vaccination among HWs conducted by Dini et al. points to much greater vaccine acceptance among physicians than among nurses [33]. Efforts to boost vaccination coverage in various occupational groups should be individualized as the reported hesitancy reasons differ between them. In the meta-analysis by Nowak et al. nurses’ vaccination hesitancy was based on the belief that the vaccine was ineffective or that influenza was not a severe disease and influenza vaccination was more important for “older nurses” [34]. Dubov and Phung identified four types of HWs who do not, as a rule, get vaccinated against influenza: the unaware, who do not believe influenza is a severe disease; the non-believers, who do not believe in the effectiveness of the vaccine; the unmotivated, who fear side effects and complications; and the uninterested, who do blame their hesitancy on poor availability of vaccines [35]. We included each of these elements in our study and observed that general attitudes towards influenza vaccination among HWs were rather positive, but nurses were less likely to agree with statements claiming that influenza vaccine was safe and effective or that medical staff was an influenza risk group.

Surprisingly, in our study people under 40 years old were more likely to be vaccinated against influenza than those over 40. This contrasts sharply with studies from both Poland and other countries. In another study conducted among Polish pediatricians, a greater willingness to vaccinate annually was reported by older physicians, though over 90% of the study group was >40 years old, which reflects the fact that the average age of pediatricians in Poland is close to 60 years old [25]. The older age also positively correlated with attitudes toward influenza vaccination in a general Polish population [21]. In a study conducted in Italy, older or immunocompromised HWs were found to get vaccinated against influenza more frequently [36]. Similar conclusions were drawn by To et al., who found that people over 50 are more likely to get vaccinated [23]. Older people more commonly have chronic conditions, consult doctors and—in some countries (including Poland)–are involved in vaccination reimbursement programs. These factors however did not prevail in our study group. We can only hypothesize that in this specific academic population with a huge subset of HWs caring for children, younger people during their pediatric education are more aware of influenza risk and the need for prophylaxis.

Several previous studies showed a link between knowledge and vaccination [37, 38]. A review of 25 studies by Lorenc et al. points to a firm belief among HWs regarding the side effects of the influenza vaccine, which undermines the incentive to get vaccinated [39]. Livni et al. found that healthcare providers, including those working in pediatrics, with better knowledge of influenza vaccination and influenza itself, were more likely to get vaccinated than those with poor knowledge [40]. A meta-analysis by Paterson et al. found that HWs being vaccinated or trained in immunization and advising patients on vaccine-related matters increase vaccine acceptance. They also found that boosting vaccination coverage among medical staff can be achieved through education [41, 42]. This is also echoed in our results, as our respondents declared unanimously that participating in training considering vaccination as well as knowledge about its benefits would convince them to get vaccinated.

Almost all HWs included in our study had direct contact with patients, and more than 60% took care for immunocompromised. In a study by Elder et al., laboratory-confirmed influenza during the H1N1 outbreak was reported in 23% of medical staff [12]. In addition, a higher percentage rate of asymptomatic infections in HWs than in the non-medical population, may further favor unwitting transmission to patients [10, 13]. At the same time, the systematic review by Li et al, shows that the higher the influenza vaccination rate among HWs, the lower the incidence rate of hospital-acquired influenza among patients. Moreover, vaccination against influenza may reduce the incidence of laboratory-confirmed influenza by 64% [14]. Therefore, vaccination against flu as a means of protecting patients is strongly justified, especially when HWs contact immunocompromised patients [15]. To improve the prognosis in immunocompromised patients, containment of vaccine-preventable infections is pivotal [43, 44].

Our study also found a significant relationship between occupation and acceptance of obligatory influenza vaccination, with particularly high discrepancy between physicians and nurses. Opinions regarding mandatory influenza vaccinations for HWs vary–with some claiming that it supports fulfilling HWs’ primary duty, which is to care for patients’ health, and others listing numerous reasons (legal, moral, educational) against mandatory vaccinations. [45, 46]. Objectively, various (full or partial) mandatory influenza vaccinations programs in HWs substantially increase vaccination coverage: in the United States and the United Kingdom the vaccination rate is above 75% and in Finland it exceeded 90% [17, 18, 47–52]. Higher vaccination rates not only improves protection of HWs and patients but also builds up the trust in health care and strengthen the role of vaccines in prophylactics [53]. Moreover, mandatory influenza vaccinations also have substantial economic value. The authors of an analysis from Italy estimated that an increase in vaccination coverage among HCWs of 10% could save €1,301,394.93 in terms of social cost and contribution to the sustainability of healthcare systems in general [54]. However, our results underline low acceptance of mandatory vaccinations among nurses, which is suggestive of that such program should be combined with other persuasive interventions in this particular group, as it was suggested by Schumacher et al [26].

In our study more than 50% of respondents declared the will to get the influenza vaccination in 2020/2021 season, compared to 25% declared as being vaccinated against infleunza a year before. Also, more than 34% of responders totally agreed with statement that influenza vaccination was essential in the prevailing COVID-19 pandemic, which is coherent with the results of other studies. In several different countries, including Poland, an increase in influenza vaccination rate was reported during the COVID-19 pandemic in the group of people over 65, with the highest increase recorded in Spain (up 13% from the previous season) and the lowest in the Philippines (up 3.0% from 2019–20 season) [55]. In Italy, the influenza vaccination rate among HWs in the 2018/2019 season was 3.7%, in the 2019/2020 season– 15.7%, and 53.4% during the COVID-19 pandemic [56]. The literature shows that the COVID-19 pandemic has increased influenza vaccination coverage among medical staff, but this effect might likely be transient [57, 58].

### Study limitations

The information about the vaccination status of HWs is based on self-report, and we consider it a limitation of our study.

Our study was conducted in academic hospitals of a capital city, which limits its generalizability–HWs employed in those hospitals are likely younger, still receiving medical education, and half of them care for children. However, the attitudes of academic society is particularly important to study, as university workers provide education to many HWs in the country.

The additional measures to promote and provide free and on-site vaccinations to HWs of our pediatric hospital in 2020/2021 season might also influenced the responses to our survey.

Another factor probably influencing the attitudes of our respondents was the COVID-19 pandemic, which provoked discussion about the importance of influenza vaccination.

## Conclusions and practical considerations

Among HWs in academic hospitals, males, people <40 years old, physicians and those working in children’s hospital are more likely to get vaccinated and their attitudes towards influenza vaccination are more positive. Of those less likely to get vaccinated, people >40 years old and nurses could be effectively persuaded by free and on-site influenza vaccination. Moreover, free access to vaccination is the strongest motivator to get vaccinated among all HWs. The attitudes towards mandatory influenza vaccination differ sharply among HWs–while physicians are ready to accept it, nurses are not.

## Supporting information

S1 File Contains translation of the whole survey and all the supporting figures. (DOCX)
