# Supplementary material for: Influenza vaccination from the perspective of health care workers at university hospitals
Source: PLoS One. 2023 Jul 21;18(7):e0288470. doi: 10.1371/journal.pone.0288470 (PMC10361510; doi:10.1371/journal.pone.0288470)
Supplement: S1 File — (DOCX) [file pone.0288470.s001.docx]

**Dear Respondent,**

**I am conducting research among the medical personnel of the University Clinical Centre of the Medical University of Warsaw. Its aim is to inquire about your attitudes towards influenza vaccination. Your participation in the survey is fully anonymous. Thank you for choosing to participate in this study.**

**The survey will take approximately 3 minutes to complete.**

**DEMOGRAPHIC DATA**

**Sex**

female  mężczyzna male

**Occupation**

doctor  pielęgniarka  nurse  ratownik medyczny  pharmacy worker  pracownik laboratorium  laboratory worker

administration worker  pracownik apteki paramedic pracownik aptekipsychologist pracownik apteki radiology/EEG technician

pracownik apteki medical receptionist pracownik apteki medical secretary pracownik apteki dietician pracownik apteki physiotherapist pracownik apteki other

**Age**

18-30 years 31-40 lat  31-40 years  41-50 lat  41-50 years  51-60 lat  51-60 years  ≥61 lat >60 years

**Years of service**

0-1 years  2-5 years  6-10 years  11-20 years  >20 years

**Workplace**

Central Clinical Hospital  Children's Clinical Hospital

The Infant Jesus Clinical Hospital

**I. Please select one answer for each question:**

1. Do you have contact with patients at work?

a.  tak yes

b.  nie no

1. Are there any immunosuppressed patients (with reduced immunity) in your workplace?

a.  tak yes

b.  nie no

1. Have you been vaccinated against influenza in the 2019/2020 season?

a.  tak yes

b.  nie no

c. nie not sure

**4.** Are you planning to get vaccinated against influenza in the 2020/2021 season?

a.  tak yes

b.  nie no

c.  not sure

**5.** Would you get vaccinated if FREE influenza vaccinations were available at the University Clinical Centre of the Medical University of Warsaw in the coming season?

a.  tak yes

b.  nie no

c.  not sure

**II. Please indicate the extent to which you agree with the following statements, where:**

1- fully disagree

2- rather disagree

3- slightly agree

4- partly agree

5- largely agree

6- almost fully agree

7- fully agree

**1. In my opinion:**

1. It is an ethical duty for everyone who comes into contact with patients to get vaccinated against influenza

1 22  33  44  55  65  75

1. vaccination effectively protects against influenza

1 22  33  44  55  65  75

1. vaccination effectively protects against influenza complications

1 22  33  44  55  65  75

1. medical staff in the influenza risk group should get a vaccine

1 22  33  44  55  65  75

1. complications after influenza vaccine are rare and should not discourage vaccination

1 22  33  44  55  65  75

1. influenza vaccination is particularly important in the context of the ongoing COVID19 pandemic

1 22  33  44  55  65  75

**2. What would motivate you to get vaccinated against influenza? (*if you already get vaccinated against influenza, what do you think could convince non-vaccinated healthcare professionals to get vaccinated against influenza):**

1. introducing the obligation to vaccinate against influenza among medical personnel (doctor, nurse, paramedic, laboratory worker, administration worker, radiology technician, medical receptionist)

1 22  33  44  55  65  75

1. influenza vaccination in the workplace

1 22  33  44  55  65  75

1. influenza vaccination during working hours

1 22  33  44  55  65  75

1. attending a training course on influenza vaccination

1 22  33  44  55  65  75

e. free access to vaccination for hospital medical personnel

1 22  33  44  55  65  75

f. being fully informed about the benefits of influenza vaccination

1 22  33  44  55  65  75

**3. In my opinion, the most effective ways to prevent influenza virus infection include:**

1. mouth and nose protection mask

1 22  33  44  55  65  75

1. washing hands

1 22  33  44  55  65  75

1. influenza vaccination

1 22  33  44  55  65  75

1. avoiding contact with those infected

1 22  33  44  55  65  75

1. consuming garlic

1 22  33  44  55  65  75

1. using inosine preparations (e.g. *Neosine*)

1 22  33  44  55  65  75

1. daily intake of vitamin C

1 22  33  44  55  65  75

**Figure 1**. Vaccinated/unvaccinated vs. contact with immunocompromised patients (N=950)

Note. V – vaccinated, NV – nonvaccinated, DR – do not remember

**Figure 2.** Influenza vaccination vs. contact with immunocompromised persons by type of profession (N=950)

Note. V – vaccinated, NV – nonvaccinated, DR – do not remember

**Figure 3.** Influenza vaccination vs. contact with immunosuppressed persons by workplace (N=950)

Note. V – vaccinated, NV – nonvaccinated, DR – do not remember

**Figure 4.** Motivation to get vaccinated against influenza (N=950)

Note. TD – totally disagree, D – disagree, RD – rather disagree, NADA – neither disagree nor agree, RA – rather agree, A – agree, TA – totally agree

**Figure 5**. Introduction of mandatory influenza vaccination among medical personnel as a motivator for vaccination by profession. (N=950)

Note. TD – totally disagree, D – disagree, RD – rather disagree, NADA – neither disagree nor agree, RA – rather agree, A – agree, TA – totally agree

**Figure 6.** Provision of flu vaccination in the workplace as a motivator for vaccination by profession. (N=950)

Note. TD – totally disagree, D – disagree, RD – rather disagree, NADA – neither disagree nor agree, RA – rather agree, A – agree, TA – totally agree

**Figure 7.** Getting a flu vaccination during working hours as a motivator to get vaccinated by profession (N=950)

Note. TD – totally disagree, D – disagree, RD – rather disagree, NADA – neither disagree nor agree, RA – rather agree, A – agree, TA – totally agree

**Figure 8.** Participation in training providing information on influenza vaccination as a motivator for vaccination by profession (N=950)

Note. TD – totally disagree, D – disagree, RD – rather disagree, NADA – neither disagree nor agree, RA – rather agree, A – agree, TA – totally agree

**Figure 9.** Free access to vaccination for hospital staff as a motivator to get vaccinated by profession (N=950)

Note. TD – totally disagree, D – disagree, RD – rather disagree, NADA – neither disagree nor agree, RA – rather agree, A – agree, TA – totally agree

**Figure 10.** Having complete information about the benefits of influenza vaccination as a motivator to get vaccinated by profession (N=950)

Note. TD – totally disagree, D – disagree, RD – rather disagree, NADA – neither disagree nor agree, RA – rather agree, A – agree, TA – totally agree
